# Domestic Foal Weaning: Need for Re-Thinking Breeding Practices?

**DOI:** 10.3390/ani10020361

**Published:** 2020-02-23

**Authors:** Séverine Henry, Hrefna Sigurjónsdóttir, Aziliz Klapper, Julie Joubert, Gabrielle Montier, Martine Hausberger

**Affiliations:** 1 Univ Rennes, Normandie Univ, CNRS, EthoS (Éthologie Animale et Humaine)—UMR 6552, F-35000 Rennes, France; aziliz.klapper@etudiant.univ-rennes1.fr (A.K.); julie.joubert@etudiant.univ-rennes1.fr (J.J.); gabrielle.montier@etudiant.univ-rennes1.fr (G.M.); martine.hausberger@univ-rennes1.fr (M.H.); 2 Faculty of Subject Teacher Education, School of Education, University of Iceland, Stakkahlíð, R105 Reykjavík, Iceland; hrefnas@hi.is

**Keywords:** horses, dam-offspring relationship, artificial weaning, spontaneous weaning, welfare

## Abstract

**Simple Summary:**

Under domestic conditions, most foals are artificially weaned before the time of natural weaning, usually at 4 to 7 months of age. Artificial weaning is recognized as a major source of stress that can also lead to long-lasting deleterious effects. This common practice seriously impairs the welfare of foals. To date, there is still little data on the natural process of weaning and the immediate consequences for both partners. After reviewing the current scientific knowledge on this subject, we present the results of a longitudinal study carried out around the weaning period on mares and their foals kept under naturalistic conditions. We found that most foals were weaned spontaneously between 9 and 10 months of age, and overall, that natural weaning induced no stress response in either partner and no sign of rejection from the dam. The findings of this study can provide new insights on the management of weaning in breeding farms, and even lead to reconsider what is commonly practiced.

**Abstract:**

Artificial weaning is a standard practice known to be one of the most stressful events in a domestic foal’s life. Research has mainly focused on ways to alleviate weaning stress. However, there is still a need for more detailed research on what should constitute best practices with respect to animal welfare. The aim of this review is to address this issue by examining the natural weaning process. We first provide an overview of the scientific literature on the natural temporal dynamics of the dam-offspring bond in horses: it is to be noted that the natural process of weaning is little documented, individual variations have been poorly investigated and immediate effects of weaning on the mare–foal relationship remain unexplored. To partly address these gaps, we performed a study around the weaning period on 16 mare–foal pairs kept with minimal human interference. Most foals were weaned spontaneously when 9-10 months old, with individual variations mainly due to the conception rate of mares. Natural weaning induced no stress response in either partner and was performed without clear signs of rejection by the dams either just before or after. We lastly open up the discussion on the need for rethinking weaning practices under domestic conditions.

## 1. Introduction

Under domestic conditions, most foals are artificially weaned prior to the time of natural weaning, usually between the age of 4 and 7 months [1,2]. The breaking of the mare–foal bond (associated with the disruption of milk intake) is usually abrupt, by placing the foal out of sight and sound of its dam, sometimes more gradual, but in all cases, takes place when the foal is still closely bonded to its mother [1,2,3]. In addition, the foal generally undergoes additional nutritional, social and environmental challenges. Even though the precise origin of early artificial weaning is unclear, it is important to go back to the sources of such a practice. In the second half of the 19th century, several studies highlighted two main results that probably led to the routine practice of early artificial weaning. Firstly, it has been found that the maternal milk production decreases sharply by the third month of lactation [4] and secondly, that the nutritional requirements of 3–4 month-old foals exceed the level of nutrients available from maternal milk [5,6]. From that point, early weaning may have been considered the best decision to make to optimize the physical development of domestic foals. This practice became rapidly widespread in professional breeding farms followed by non-professional breeders of one or two mares. Nowadays, there are many practical, economical and safety reasons to proceed to the early weaning of foals, such as allowing for an early marketing of foals, switching the foal’s attention from the mother to humans [7,8], facilitating the management of the foal’s nutritional intake without the mare interfering, or even optimizing the subsequent reproductive efficiency of the mares by limiting the potential negative impact of a prolonged nursing. Some of the reasons are based more on habits and tradition, perhaps even on false beliefs, and clearly not on the prospects for improving the welfare of domestic foals. 

While the impact of artificial weaning on mares has not yet been properly examined [9], it is now well admitted that artificial weaning is one of the most stressful events in a foal’s life [1,3,10]. The well-known behavioral responses to early weaning (e.g., increased long-distance whinny calls, increased active locomotion and eliminative behaviors) and the associated risks of injury, typically peak within the first two post-weaning days [2,11,12,13]. However, other behavioral changes such as altered feeding and sleeping patterns, aggressiveness, suspension of play and redirected suckling towards peers stemming from frustration, may be observed for much longer periods [14,15,16,17,18]. Weanlings also experience elevated glucocorticoid (stress hormones) levels, changes in heart rate, as well as a decline in growth rates [1,3,12,13,17,19,20,21]. Most of these behavioral and physiological manifestations are more intense in the first post-weaning days and vanish within two-three weeks. Artificial weaning results also in chronic problems (which are more difficult to link to artificial weaning by foals owners). Thus, stress hormones lead to a subsequent decrease in immune response [20,21,22]. They have also recently been reported to have a negative impact on the maturation of the gut microbiota which can lead to the potential growth of harmful microorganisms such as *Escherichia coli* [21], while, in other species, recent studies emphasize the key role played by intestinal microbiota in the long-term health of the host [23,24]. Numerous scientific studies also point out, in foals [2,3,25,26], as in other domestic or captive young mammals [27], that early weaning is a major cause of stereotypic behaviors and thus of animal welfare impairment. Thus, a 4-year prospective study performed on 225 young horses (70% were weaned between the age of 4 and 6 months) revealed that crib-biting was initiated by 10% of the foals at a median age of 20 weeks (i.e., 5 months), 30% showed excessive wood-chewing (i.e., lignophagia) by 30 weeks of age (i.e., 7.5 months) and 7% suffered from locomotor stereotypies by the age of 64 weeks (i.e., 1.5 years) [25]. Although this has not been demonstrated to date in weanlings, it is worth noting that most studies found altered learning abilities in adult stereotypic horses as compared to healthy horses [28,29,30]. According to Wolter [31], early artifical weaning can also lead in the long term to other disorders, especially in future breeding mares who could later be less fertile and produce weaker, slower-growing foals with a weaker skeleton. Some proposals to limit the distress induced by weaning have been put forward [1,3], such as progressive retrieval of the mares [13], repeating separations [32,33] and introducing adults in groups of weanlings [13,17]. However, few studies investigated the importance of the timing of artificial weaning [1,3,18,34] and, overall, none of the previous studies questioned the practice of artificial weaning.

Without human interference, the situation is of course dramatically different. It is often reported that weaning takes place gradually over several months through the joint initiative of the dam and the foal, and that foals are usually not weaned before the age of 9-11 months or until shortly before the birth of the next foal. In addition, “natural weaning” only implies a nutritional aspect, as the close dam-offspring bond remains afterwards for much longer time [35,36]. Beyond these general statements, the weaning process (such as the gradual process of maternal rejection or distancing between the mare and its foal over time) is however still relatively unknown as few scientific studies have been done at the later stages of the lactating period (i.e., after the first 6 months) and the potential factors of variation in the weaning process remain poorly documented [37,38,39]. After reviewing the scientific literature on the temporal dynamics of the dam-offspring bond in horses living in naturalistic conditions, we present the results of a study conducted in Iceland on mare–foal dyads (n = 16) kept outdoors in stable social groups with limited human interference (i.e., no intervention in foaling and no artificial weaning). We believe that better knowledge of the natural weaning process can provide new insights on the management of weaning under domestic conditions, and even lead us to reconsider such routine practice. 

## 2. Spontaneous Weaning In (Semi-) Natural Conditions: An Overview of The Scientific Literature

### 2.1. Social Structure, Reproduction and Gestation

In (semi-) natural conditions, horses form stable social networks. The basic social unit is the family group (harem) consisting generally of two to six individuals, including a breeding stallion (sometimes two), 2–3 breeding mares and their sexually immature offspring (until the age of 2–3 years) [36,40]. The reproduction is seasonal, with a peak in breeding and foaling from late winter to early summer when nutritional and weather conditions are more favorable for the lactating mare and the growing foal [41]. The average gestation period is just over 11 months (340 days). In domestic horse breeds, the normal gestation length (i.e., resulting in healthy foals) is known to range from 315 to 387 (or even up to 404 in some breeds) days [40,42], thus showing a large variation (with an amplitude of variation of almost 3 months) that is quite unusual among mammalian species. The factors involved in gestational length may be environmental (e.g., month of conception or parturition, weather and nutrional conditions, climate), fetal (e.g., sex and breed of the fetus), maternal (i.e., maternal age and parity, maternal lineage, nutritional status) or (to a lesser extent) related to the stallion [42]. In general, gestation periods are longer in young and primiparous mares, in mares that conceive earlier in the year, are in poor nutritional condition or are carrying colts. In addition, there is a great individual variation in the length of pregnancy partly due to genetic factors (e.g., maternal lineage, breed). Mares are monotocous and have generally one foal at a time [40,41]. Although twin pregnancies can probably occur occasionally as in domestic situations, they also probably result in abortions, stillbirths and neonatal mortality [43], which may explain why, to our knowledge, they have never been reported under (semi-)natural conditions. Usually, foaling takes place some distance away from the herd [37,40,44]. It has been reported that mares who foal for the first time (i.e., nulliparous mares) are less likely to withdraw from the social group [40].

### 2.2. Dynamics of the Mare–foal Relationship and Weaning Process

In the first few days after foaling, mares keep their foals at their side and greatly limit direct contact between their newborn foals and other horses [44,45], thus allowing a selective mother-offspring bond to develop soon after birth [46,47]. This close protection gradually reduces after the first days. During the first month of life, foals are however largely dependent on their dams, as maternal milk represents their only source of nourishment. Foals suckle on average 4-7 times per hour (i.e., about 60 times a day) which represents about 8% of the time-budget of foals, and mares and foals are within 5 meters of each other most of the time (more than 90%) [34]. Over time, mare–foal relationships change with a gradual reduction in the frequency of suckling combined with foals’ increased consumption of solid foods and increased social independence from dams. 

#### 2.2.1. Dietary Independency: A Gradual Transition from a Milk Diet to Solid Food 

As the age of foals increases, the duration and frequency of suckling decrease, with a sharp drop between the first (~8% of the time-budget) and second month of life (~3%). Then, the time devoted to suckling (about 1–2%) varies very little: foals suckle on average once per hour until 7 months of age and about once every two hours from 8 months until weaning [37,38]. In most cases, there are no significant differences between colts and fillies in the rate of suckling, duration of suckling bouts and energy intake from maternal milk [37,39,48,49], nor are there significant differences in the proportion of suckling bouts that are ended by the mother and the proportion of unsuccessful suckling attempts [48]. In addition, no significant difference between primiparous and multiparous mares has been reported [37,49], nor of non-pregnant and pregnant mares [50]. During most of the lactation period (i.e., from the first month to the seventh), foals terminate most suckling bouts (at least 50%). From the eighth month of lactation (as compared to earlier stages), termination of suckling bouts by dams increase and dams tend to exhibit more signs of rejection during their foals’ suckling initiatives: thus, they may reject suckling attemps or terminate suckling bouts by moving away, blocking access to the nipple by flexing a hind leg, or more usually at that stage by displaying threats or other mild agonistic behaviors [37,38,40,48,49,51]. This increase in maternal rejection at the end of lactation has been considered an important step in the weaning process [38,40], although it has not been systematically observed [37,52] and could rather be affected by the nutritional status of the mare [38].

In parallel, milk composition and milk yield are gradually changing over time [53]. During the first month, the composition of the milk gradually changes: the fat and protein content decreases, as do the calories, which probably helps foals start to diversify their diet. However, milk production increases during the first four to eight weeks of lactation until it peaks at 8 to 12 liters per day (2 to 3.5kg milk per 100 kg body weight per day) before slowly decreasing, resulting in lower energy costs for the mare. The composition of the milk also changes during the first month: the fat and protein content decreases, as do the calories, which may result in an increased motivation of the foal to seek alternative forms of nutrition. From the age of 2-3 weeks, foals begin to ingest solid foods and diversify their diet while continuing to suckle. Between 1 and 4 months, the time devoted to grazing in the daily time-budget increases from 7% to 25%, at 6 months it reaches more than 40%, and at 10 months 60% which corresponds to the adult time-budget [40]. Currently, little is known about the development of dietary selectivity in foals. Of all the mechanisms suggested, transmission through maternal milk cannot be excluded and coprophagy (i.e., ingestion of feces, particularly those of dams, that is reported from the age of 2 weeks to 3 months) has been considered to allow the transmission of food preferences [54,55,56]. In addition, the dams probably play a key role as they constitute the first social models and are thus known to greatly influence the behavior of their foals [57,58]: young foals are often reported to graze with their dams at their side which could allow them to observe the appropriate food items to select (type of plant, part of the plant, state of development of the plant, etc.) [59]. The transition from a milk diet to a solid diet is gradual, allowing a progressive adaptation of the foals’ digestive system. While foals are born with a sterile gut, microbial colonization begins the first day of life (even maybe in utero) [60,61,62]: thus, foals are gradually colonized by numerous bacterial species with which they come into contact via the maternal body (e.g., vaginal microbiota of the mare, udders…) and environment (e.g., inoculation by ingestion of feces is common from the age of 2 weeks) [54,63]. This allows the establishment of the gut microbiota necessary for fiber digestion [31,60,63]. According to the study by Faubladier et al. [60], cellulolytic bacteria can be detected in the feces of foals from the third day of life, and at 2 months of age foals have acquired the ability to digest plant fibers. The development of the digestive function also reflects the increasing consumption of solid foods by foals. This slow adaptation to adult feeding patterns will prepare foals for weaning (i.e., the cessation of suckling) and would limit post-weaning disturbances such as digestive disorders or growth retardations [31].

#### 2.2.2. Social Emancipation: A Progressive Widening of the Social Network 

Another component of the weaning process is the social emancipation of the foal. Longitudinal studies show that foals, as they grow older, gradually move away from their mothers: they remain close (i.e., within 5 metres) 70% and then 40% of the time at the age of 3 and 6 months, respectively, but at 9 months they still remain within 5 metres of their mothers 20% of the time. Most changes in mare–foal distances are primarily due to the foals’ initiatives, but, when foals are lying down, the dams stay in their close proximity by grazing in a circle around or resting standing right beside their foals [39,59]. This particular behavior, called “the recumbency response”, can be observed until the foal reaches the age of 3 to 4 months. Once more, no difference has been reported according to the gender of foals [39,40]. In parallel, from the age of 3-4 weeks, social interactions with other individuals than the dam, and more particularly peers, become more numerous [37,59]. This phase of socialization, which peaks at 2-3 months, is characterized by a high frequency of social play and, to a lesser extent, mutual grooming between foals, and by an increase in snapping (i.e., juvenile submissive behavior directed at adult horses) towards adults [59]. From the age of 4 months, foals enter a period of gradual emancipation with the appearance of adult behaviors [59]. However, beyond this period, foals and their dams remain mutual preferred spatial partners and even at the age of 1 year, foals spend less than a third of their time more than 45 m from their dams [37,64].

#### 2.2.3. Conclusion 

In conclusion, weaning occurs over several months by a gradual increase in the mare–foal distance, a progressive decrease in suckling frequency with a change to a more varied diet, and the development of a larger social network. Interestingly, several studies show that changes in mare–foal distance, but also suckling activities, are mainly due to the foal’s initiatives, thus demonstrating the active role also played by foals in the weaning process [40,59,65,66]. In addition, some scientific works highlighted behavioral variations (e.g., frequency of social play, distancing from the dam, suckling frequency…) among foals of the same breed from the youngest age despite homogeneous living conditions [65,66,67]: some foals stay, for example, very close to their mothers, suckle more frequently and interact less with peers than their same-aged counterparts. Such individual variations can be related to genetic (e.g., sire effect) and/or experiential (e.g., early adverse experiences) influences [59,65,66,67]. For instance, foals from different sires showed at 3 months of age some differences in the time spent suckling, playing and in dam–foal distance [65].

### 2.3. Natural Foal Weaning and Factors of Variation

#### 2.3.1. Age at Weaning

In (semi-) natural conditions, spontaneous weaning of foals is thought to be mainly initiated by the dams when their foals are about 9 to 11 months old [37,38,39,40,59]. In the study of Tyler [37], most foals were weaned around the age of one year, a few days or weeks before their dams give birth to a new foal. Weaning age can vary considerably: in the study of Berger [68], 79% of foals were weaned before the age of 9 months, 6% between 9 and 12 months, and 15% at a later age. However, it is not uncommon for yearlings, or even two- and three-year-old young horses, to continue suckling regularly their dams in addition to the foal of the year [37,39] [Sigurjónsdóttir and Hausberger, personal observations]. The duration of the dry period (i.e., the time between two successive suckling periods) is highly variable (ranging from 0 to 23 weeks): although the production of milk is not necessarily interrupted between two successive foals [37], the dry period lasts most often between 10 and 16 weeks [38].

#### 2.3.2. Factors of Variation

As seen previously, the age at which the foal is weaned, may vary considerably. While these differences do not appear to be sex-dependent [37,38,68,69], a few factors of variation related to the dam have been identified or mentioned:

- The reproductive status of the mare (i.e., pregnant or non-pregnant mare) has been considered an important factor determining the time of weaning [37,38]. Pregnant mares tend to wean their foals on average 3-4 months before giving birth to the next foal, at the time of gestation when the energy costs become significant: indeed, the fetal growth, which is at first slow, increases exponentially during the last trimester of pregnancy and the nutrient requirements of the fetus become significantly greater. Conversely, non-pregnant dams continue to nurse their foals for a longer time (i.e., up to a year and a half, or even longer). In addition, it is worth noting that, in the event of foal death, some mares nurse their older offspring much longer (up to their third year) [37].
- The previous breeding status of the mare (i.e., mare with or without a yearling) can also be at stake: mares with a yearling and a foal of the year tend to wean the latter at about 8.5 months, while mares who did not have a foal in the previous year can wean the foal of the year at a later age (at about 16 months) [68].
- The maternal parity (i.e., primiparous/multiparous) can also influence the age of foals at weaning: thus, some primiparous mares (i.e., raising their first foals) nurse their foals over a longer period (on average of two extra months) as compared to multiparous mares [38]. This tendency, however, has not been systematically reported [37,49].
- Other potential factors affecting the timing of weaning include the availability of food resources, weather conditions or maternal body condition.

Scientific data on weaning age and/or lactation duration are quite limited. While further studies are needed to confirm the effects of the above factors, other factors, such as the change in milk composition, the impact of natal group composition (e.g., the presence of other young horses, related or not, of the stallion, etc.) or the active role of foals in the weaning process, have received little or no attention to date. One has to consider that the own behavioral characteristics or personality of foals could also be involved in determining the age of weaning. As mentioned previously, foals exhibit early behavioral differences, especially in their relationship with their dams (frequency of suckling, spatial proximity) that may reflect differences in terms of emotionality or need for reassurance by seeking maternal contact. These differences are therefore likely to have an impact on the temporal course of post-natal development and emancipation of foals [70].

#### 2.3.3. Cessation of Nursing, but no Rupture of the Dam-Foal Social Bond

In (semi-) natural situations, weaning refers only to the gradual process of foals transitioning from a liquid-based milk diet to solid food. The close relationship between the mare and her offspring, even when the mare has new foals, continues until the youngster becomes sexually mature and leaves its natal group: at dispersal, most young mares move to reproductive units, whereas most young stallions disperse to groups of bachelor stallions [40,68,71]. Dispersal of young horses from their natal group and as a consequence the rupture of the dam-offspring bond thus occur around the age of 2 to 3 years [40]. A study performed on 12 one- and two-year-old Przewalski horses demonstrated that juveniles do not associate randomly with social partners of their natal group, but choose more often certain social partners compared to others as their nearest neighbors and/or most preferred interaction partners [64]. While social preferences of young horses for interaction partners change with age, the mother remains the most preferred spatial partner regardless of the age and sex of juveniles. Thus, under domestic conditions, it is clear that the rupture of the mare–foal social bond at 4-7 months is far too early. 

## 3. A Study on Spontaneous Weaning in Domestic Foals

In horses, the temporal dynamics of the mother-offspring bond during the late stage of the lactation period (i.e., when foals are older than 6 months of age) remains nowadays poorly documented because studies are few in number and/or mostly based on discontinuous observations. It should be noted that the natural process of weaning and individual variations have been little studied, while the immediate effects of weaning on the mare–foal relationship and behavior of both partners remain unexplored. To partly address these gaps, we performed a study around the weaning period on Icelandic mares and their foals, kept outdoors with minimal human interference. In some places in Iceland, horse management practice provides a unique opportunity to observe such situations under semi-natural domestic conditions, with foals allowed to stay with their dams until they are at least 1 or 2 years old. First, we focused on the age of foals at weaning and potential factors of variation including the age, parity, conception rate and body condition of mares, as well as the date of next foaling and the sex of both the current and upcoming foal (as all mares were again pregnant). Second, we examined, retrospectively, whether the mare–foal relationship, as well as the behavior of mares and foals, exhibited changes just prior to and/or just after the weaning of foals.

### 3.1. Subjects and Study Site

This study was conducted on 16 Icelandic mare–foal dyads living in three stable social groups, composed of other mare–foal dyads and yearlings (Table 1). The mares, aged 7 to 23 years old, were all multiparous with parity ranging from 2 to 13 (an average of 4.5 prior foals) and again pregnant. All the mares were in the group with their foals of the year and their yearlings. The foals, 9 females and 7 males, were all born between May and July, and were issued from 11 sires. Horses belonged to the Farm at Holar University College, Iceland (study groups 1 and 2) and to a professional breeder (study group 3) at the farm Kalfstadir, close by Holar. In both sites, all the groups were maintained all the year round in semi-natural settings (large pastures with natural resources, stable social groups). All horses from the same social group have been kept together at least for a year prior to the start of the study. Animals were supplemented with hay (in winter only) and salt. The water supply came from natural streams and sometimes in winter by the ingestion of snow. Human interference was very limited outside of the distribution of forage and prophylaxis care: foaling took place outdoors without human assistance, juveniles were left with their mothers up to the age of 2 years. In both sites, young males are usually removed from the natal group around the age of 12 months, either temporarily or permanently, respectively to undergo castration or prevent them from covering females.

### 3.2. Observations

Daily observations were conducted from February to May, when most foals were over 6 months of age. Special attention was paid to various variables commonly used to assess the quality of the mother-offspring relationship in horses. These include mare–foal distances, choice of the dam as preferred neighbor, frequency and quality of mare–foal interactions including suckling activities [37,65,66]. All observation sessions took place during the daytime period between 10:00am to 5:00pm, with 3 to 6 observation days per week and per study group (depending on the weather conditions, e.g., snowstorms, temperature, etc.). Observation periods changed every day following a rotation schedule. Observations were tape-recorded and transcribed later. Three observers were involved in data collection. Reliability was controlled using the kappa coefficient of Cohen [72] that rated at k = 0.95.

All behaviors of foals and their dams were continuously recorded (“focal sampling” [73]) for 5 minutes, 6 times a week (30mn. per individual per week), for details on frequency (occurrences per hour) and type of behaviors expressed by each foal and each mare. The following behavioral items were recorded: locomotion, exploration (e.g., sniffing the ground), grazing, feeding (e.g., hay), drinking, resting standing or lying down, solitary play (including manipulation of an object and locomotion play), self-grooming and social interactions with mother/foal and/or other social partners. Interactions recorded were as follows [40]: (a) social investigations (e.g., sniffing through naso–nasal, nasal–body or naso–genital contact); (b) affiliative interactions (e.g., mutual grooming, other physical contacts, social play); and (c) agonistic interactions (e.g., threats to bite or kick, bite, kick). 

Suckling activities and associated behaviors were also recorded each time they were observed in the group, specifying the identity of the dyad concerned (“all occurrences of behavior") [73]. Each week, two observation sessions of 1.5 hours each (as foals suckle on average once per hour at this age) were carried out. During these sessions, we noted the number of suckling attempts (i.e., foal suckling initiatives rejected and terminated by the dam, duration < 5 sec.), the number of (successful) suckling bouts, which partner (dam or foal) terminated the suckling bout and, if it was the dam, the associated behaviors (e.g., distancing from the foal, threat to bite…) were recorded.

The behavior of each mare and each foal, the mare–foal distance, as well as the identity and distance to the nearest neighbor of both partners, were recorded, using the “instantaneous scan sampling" method [73]. Scans were performed every 5 minutes for an hour, three times a week (36 scans per week). Distances were measured in horse body-lengths and six distance categories were used: [0] (i.e., contact), ]0;1], ]1;5], ]5;10], ]10;20], more than 20 “horse body lengths" [59]. Six social categories were used: [mother or foal of the focal individual], [other mares], [other foals], [related yearling], and [other yearlings]. Scan sampling yielded three types of data: (1) time (in %) devoted to the different behavioral items (time-budget); (2) time (in %) spent at different distances to the dam (or the foal) by the foal (or the dam) (dam-foal spatial relationship); (3) time (in %) spent near different neighbors by the foal (social preferences).

Lastly, the body condition of mares was scored monthly following the method of Arnaud et al. [74] (in addition to the follow-up carried out by the breeders). The score ranged from 0 (emaciated) to 5 (obese) with a score of 3 corresponding to an optimal body condition.

### 3.3. Analysis

As data were not distributed normally, non-parametric statistical tests were used [75]. The statistical tests were performed using Statistica® 13 (Statsoft, Tulsa, USA). All means are given ± SE (standard error).

Two main types of analyses were performed: (1) we first investigated the age of foals at weaning and the potential factors of variation (for all study groups); (2) we retrospectively compared the behavior of both partners during the two weeks prior to weaning to that scored in the two weeks following weaning (only for study groups 1 and 2). 

Weaning age was defined as the age at which the foal was last observed performing nutritive suckling. We also determined the duration of the dry period (i.e., the time from the last suckling of the current foal until the birth of the next foal) by recording the date of birth of the next foal. We calculated the coefficients of variation (CV), which represent the ratio of the standard deviation to the mean, to examine individual variation. Several factors of variation have been examined: the age, parity, conception rate and body condition of mares, as well as the date of next foaling and the sex of both the current and upcoming foal. Mann–Whitney U-tests were used to compare two independent samples (e.g., sex differences), while Spearman tests were used for correlations.

In order to examine whether weaning induces or reflects a clear change in the mother-offspring bond, we compared the behavior of both partners during the two weeks prior to weaning to that scored in the two weeks following weaning. Changes between the two time periods in mare–foal distance, social preferences and behavior of both partners were addressed using Wilcoxon signed rank tests. Preferential spatial partners were identified within family groups and based on the spatial proximity to the nearest neighbors. Preferential spatial partners of individual A were those that were more frequently the closest to A than expected by chance (partitioned chi-square goodness-of-fit test). 

### 3.4. Results

#### 3.4.1. Age at Weaning and Factors of Variation

Spontaneous weaning occurred when foals reached the average age of 9.0 ± 0.1 months. The dry period was on average of 3.2 ± 0.2 months (Table 2). There was no difference between the three study groups either for the age at weaning nor the duration of the dry period (Kruskal–Wallis: *p* > 0.1, Table 3). Considerable variations between the different mare–foal dyads were found. The age of foals at weaning ranged from 7.7 to 10.0 months, while the duration of the dry period ranged from 1.9 to 4.6 months (Table 3). Individual variations were higher for the duration of the dry period (Coefficient of variation: CV = 22.9%) as compared to the age at weaning (CV = 7.7%; Table 2 and Table 3).

The age of the foal at weaning (N_females_ = 9, N_males_ = 7; X_females_ = 8.9 ± 0.2, X_males_ = 9.0 ± 0.3; Mann–Whitney test: U = 30.5, p = 0.96) and the duration of the dry period after weaning (X_females_ = 3.1 ± 0.3, X_males_ = 3.3 ± 0.2; U = 27, *p* = 0.68) were not sex-dependant. The sex of the upcoming foal had no impact on the age of foals at weaning (weaning age: N_females_ = 11, N_males_ =5; X_females_ = 9.0 ± 0.2, X_males_ = 8.8 ± 0.4; Mann–Whitney test: U = 31, *p* = 0.73) nor the duration of the dry period (X_females_ = 3.2 ± 0.2, X_males_ = 3.2 ± 0.3; U = 28, *p* = 1). The age of foals at weaning and the duration of the dry period were not correlated with the age (Spearman: rs = 0.31, p = 0.25 and rs = -0.07, p = 0.81, respectively), the parity (rs = 0.08, p = 0.75 and rs = -0.06, p = 0.81, respectively) or the body condition (scored in the month of weaning) of mares (rs = 0.18, p = 0.57 and rs = -0.16, p = 0.59, respectively). However, it is worth noting that mares had an optimal body condition, with an average score of 3.0 ± 0.1, that remained stable over time. Only the conception rate (i.e., mean number of foals produced per year) of mares was positively correlated with the duration of the dry period (rs = 0.55, p = 0.028), while no significant correlation was found with the age of foals at weaning (rs = -0.31, p = 0.25): in other words, the more foals the mares produced on a regular basis, the longer the dry period lasted between two successive “lactation“ periods. 

#### 3.4.2. Impact of Weaning on Time-Budget and Dam-Offspring Relationship

**Figure 1:**
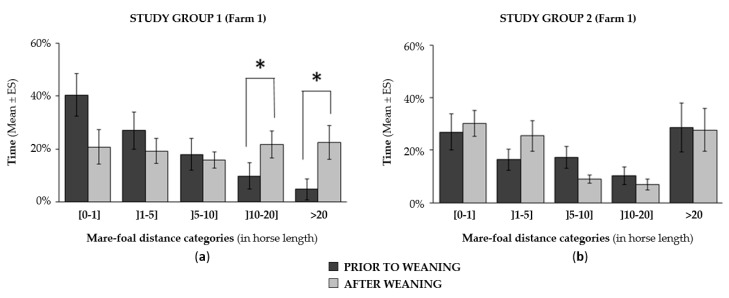
Change in the of mare–foal distance before and after weaning: (**a**) in study group 1; (**b**) in study group 2. Wilcoxon tests: * *p* < 0.05.

**Figure 2:**
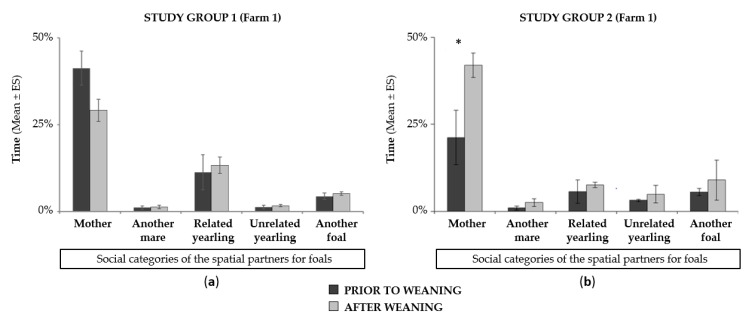
Comparison of the time spent by foals near different neighbors (social preferences) before and after weaning: (**a**) in study group 1; (**b**) in study group 2. Wilcoxon tests: * *p* < 0.05.

In the two weeks prior to weaning, mares and their foals spent the majority of time in close proximity (from 28% to 40% of the time at less than 1 horse length and from 44% to 67% of the time at less than 5 horse lengths depending on the study group; Figure 1). The dams were the most preferred spatial partner of foals among all the other individuals available within the group: foals spent from 21% to 40% of the time with their mothers as the nearest neighbor (Figure 2a,b). Similarly, the dams showed a clear preference for their foals over the other members of the group (the foal was the closest neighbor from 30% to 44% of the time depending on the study group; Figure 2a). 

Interestingly, the dams rarely displayed agonistic behaviors towards their foals (i.e., on average less than one occurrence every two hours), either during or outside of suckling activities. There was no decrease in the frequency of suckling bouts, with foals still suckling on average slightly less than once per hour (0.68 suckling bouts per hour), and they terminated most of the suckling bouts (69.2%) until the very last weeks prior to weaning.

No significant change in spatial proximity between the two partners was scored prior to and after weaning: mares and foals spent most of their time in close proximity (i.e., within one horse length; Figure 1). In addition, foals showed an equally strong preference for their dams after weaning, still choosing the mother more often as the nearest neighbor compared to other individuals in the group (Figure 2a,b). Similar findings were scored in mares in both groups (*Group 1*: X_prior to weaning_ = 44.4 ± 4.5%, X_after_ = 29.5 ± 6.1%; *Group 2*: X_prior to weaning_ = 34.1 ± 4.8%, X_after_ = 38.2 ± 4.1%; Wilcoxon test: *p* > 0.1 in all cases). Furthermore, it should be noted that no suckling attempts were observed after weaning. Finally, apart from suckling activity, no significant change in the foals’ time-budget was observed (Wilcoxon tests: 2.50 < w < 11.50, *p* > 0.1 in all cases): feeding and resting remained the two predominant activities, either two weeks before or after the weaning date. 

### 3.5. Conclusions

The age of foals at weaning, but especially the length of the dry period, varied from one dyad to another. The main explanatory factor was the conception rates of mares. It should be noted, however, that the mares in this study had a number of common individual characteristics, limiting the factors of variation: they were all multiparous, pregnant again, and in the presence of both their foals and yearlings. Contrary, perhaps to expectations, the older mares did not tend to wean their foals earlier and overall, no loss of body condition was noted over the lactation period despite severe climatic conditions (negative temperatures, presence of snow cover, etc.), the absence of food supplements other than hay and a high conception rate of mares (the majority having a foal each year). Anecdotally, in the two farms where the observations took place, the breeders never had to wean a foal artificially because the mare’s body condition required it, or only on rare occasions (e.g., once in 10 years at the Hólar University College). It is important to note, however, that on both farms, mares had access to continuous 24-hour fiber provision, which is known to improve both the body condition and fertility rate of mares [76]. It is also possible that the hardy character of the Icelandic breed may have played a role in the good maintenance of the body condition of mares. Further studies on different horse breeds are required to test for generality.

Surprisingly, the suckling frequency did not change in the two weeks prior to weaning, and mares did not express increasing rejection responses to the suckling initiatives displayed by their foals. Similarly, no suckling attempts have ever been observed in weanlings, suggesting that there was no frustration and that perhaps foals voluntarily decided to stop suckling. Finally, it is worth noting that spontaneous weaning did not induce signs of distress, as is often assumed [77], nor any change in the foal’s time-budget (apart from the cessation of suckling activities) nor in the relationship between the mare and the foal. The impact of natural weaning in terms of animal welfare obviously contrasts with that of artificial weaning commonly practiced in professional and non-professional breeding farms. 

## 4. General Discussion

Modern breeding practices generally impose strong constraints as compared to the conditions of development of foals in a more natural environment [3,10,40,63,66]. One major aspect is the early artificial weaning, which is not just a stage of diet transition but also a stage of social separation. There is increasing evidence that such a practice, although carried out on a routine basis by horse breeders, leads to short- and in some cases to long-term severe negative outcomes [1,2,3,9,10,11,12,13,14,15,16,17,18,19,20,21,22,25,31]. There is therefore a clear need to better understand the factors at stake (e.g., cessation of milk intake, immature digestive system, maternal deprivation, absence of adult models, additional changes in feeding or housing…), to improve the domestic management of weaning and animal welfare.

In this line of thought, one interesting finding of our study aimed at investigating the natural weaning process is that it induces no frustration (such as dam-directed suckling attempts or non-nutritional sucking of other foals) or distress behaviors (such as increased locomotion or aggressiveness) in either partner. These results thus suggest that the main source of stress in artificial weaning is rather the abrupt rupture of the dam–foal bond than the cessation of suckling. This would explain why gradual weaning, allowing foals to see, hear, smell, and touch their dams through a fence, but not suckle, results in fewer behavioral responses than abrupt weaning [14,19]. Similarly, a recent study that investigated the effect of a two-stage weaning method which involves separating the effects of the nutritional part (suckling) and the physical part (social bond), tends to confirm this assumption. Indeed, no significant behavioral and physiological changes were noted in foals nor in mares during the first stage when foals were only prevented from suckling their dams (by the use of udder covers) [9]. However, once mares and foals were then physically separated in a manner similar to the typical abrupt method, classical behavioral and physiological signs of distress (e.g., vocalizations, running, foal–foal aggressions, higher fecal cortisol concentrations…) were exhibited. Taken all together, these results call into question the initial assumption according to which the main aspect of the mother-foal bond is food, thus justifying the practice of artificial weaning from the age of 4 months as the mare’s milk then become insufficient for the foal’s energy requirements [4,5]. Furthermore, it has been found that artificial weaning induces a similar reduction in average daily weight gain whatever the age at which it is carried out (4.5 months or 6 months of age) [34]. To date, there is increasing evidence that other aspects relative to the dam-foal bond, such as emotional security [66,67,70] or social preferences [40,63], are at stake. This is probably one of the reasons why, outside of rare exceptions, weaning (cessation of suckling) prior to the age of 7 months almost never happens under (semi-)natural conditions [37,38,39,40,59]. In other mammal species, including humans, it is well known that young animals or infants, once the attachment bond with the mother has been established, exhibit a strong preference to stay near their mothers, even in the absence of food supply [78,79,80,81]. 

Therefore, when deciding when to artificially wean the foal, it seems important not to focus only on the age of the foal and the preparation of the feeding transition (which is most often recommended) [1,3,12,14,15,18,34,49], but also to pay more attention to individual variations in the strength of the social bond between mother and foal [3,9]. Some foals, even relatively old ones, may be less socially independent and closer to their mothers than others of the same age [65,66,67,70] and, therefore, may be more likely to respond more intensely to maternal separation [9]. For instance, a recent study by Nicol et al. [26] found that foals that are more prone to develop post-weaning abnormal behaviors, spent more time suckling, prior to weaning, than other foals. Taking into account the individual characteristics of the mare (e.g., if pregnant or not; if pregnant, her stage of pregnancy, her conception rate) also appears to be of high significance to choose the best time to perform artificial weaning based on the scientific knowledge on the natural weaning process [37]. Conversely, while weaning foals in a progressive way is often assumed to more closely imitate the natural weaning process, it is far from being the case. This weaning method consists in gradually increasing over a period of several weeks the time of maternal separation (from a few minutes to several hours) and, therefore, decreasing the possibility of suckling in foals [33]. Progressive weaning does not mimic, therefore, the natural temporal dynamics of the dam-foal bond: in line with previous scientific work [36,37], our study shows that suckling frequency does not decrease in the weeks prior to weaning, with still on average one suckling bout every two hours, and that mare–foal proximity remains stable until (and even after) weaning. The same applies to gradual weaning [14,19] or the weaning method consisting in habituating foals to maternal deprivation by separating them from their dams repeatedly before final weaning [32]. This may explain, at least in part, the discrependencies in terms of benefits obtained by these various approaches of artificial weaning [14,19,32,33].

Finally, on the basis of the scientific knowledge relative to both the deleterious short- and long-term effects of artificial weaning and the benefits of natural weaning, the question of the relevance of performing systematically early artificial weaning in domestic situations can be raised. If in professional breeding farms, natural weaning seems relatively more difficult to implement, switching to natural weaning is feasible in a number of breeding farms that own a smaller number of breeding mares (more than 80% of breeders have only 1 or 2 breeding mares). There are easy changes to make in management practices to do so, such as installing selective feeders to make sure foals have access to sufficient food or providing forage ad libitum to make sure that mares are not losing weight, especially in cases of concurrent lactation and pregnancy [60]. Maintaining foals with their dams over a longer period of time also offers the benefit to use maternal influences to facilitate the education of foals [41,42,82], to decrease the occurrence of stereotypic behaviors in stereotypic breeding mares [83], and to lower aggression within groups, horses being less agressive when in groups with foals [84]. In addition, it appears that preserving a normal ontogeny with a prolonged and secure bonding with dams is a source of protection against adult-experienced adversity: for instance, in intensive macaque breeding facilities, it has been shown that wild-born animals expressed less altered welfare (e.g., stereotypies) than captive-born animals that had been weaned early [85]. Similar questions are raised in other domestic animals, including companion (e.g., kittens and puppies) and production (e.g., calves and piglets) animals (e.g., [86,87,88]).

Re-thinking weaning practices in the domestic situation is crucial for obvious welfare reasons in both the short and long term. Further studies are needed to better identify what should constitute the best weaning practices with respect to the welfare of foals and mares (e.g., age of artificial weaning, adaptation to each mare–foal dyad, or to the body condition or reproductive status of mares). However, it also seems essential to conduct longitudinal studies to compare artificially weaned foals with foals that have been naturally weaned in order to assess all the potential consequences of artificial weaning.

## Figures and Tables

**Table 1:** Description of groups and mare–foal pairs: age and parity of mares; sex and birthdate of foals.

Items	Study Group 1	Study Group 2	Study Group 3
Group size	24	25	47
Density (number of horses per hectare)	1.10	1.32	2.35
Number of mare-foal pairs	5	4	7
**Breeding mares:**			
Mean age ± ES ^1^ [min-max] (in years)	14.8 ± 2.9 [8–23]	10.8 ± 2.2 [7–17]	13.0 ± 2.1 [9–23]
Mean number ± ES ^1^ of previous foals [min-max]	5.6 ± 2.2 [2–14]	5.5 ± 1.9 [2–11]	4.7 ± 1.4 [2–11]
**Foals:**			
Number of females/males	4/1	3/1	2/5
Birthdate [min-max]	[20/05–21/06]	[06/06–22/07]	[29/05–14/07]

^1^ ES = Error-standard.

**Table 2:** Foals age at weaning, duration of the dry period and potential factors of variation.

	FOALS		MARES			WEANING		FETUS
	Code	Sex	Age (years)	Parity	Conception rate ^1^	Age (months)	Dry period (months)	Fetal sex
GROUP 1	G1F1	F	19	2	1.0	9.2	3.5	F
	G1F2	F	23	13	0.7	9.3	2.9	F
	G1M1	M	9	3	0.7	9.1	3.0	M
	G1F3	F	8	2	1.0	8.8	3.7	F
	G1F4	F	15	8	1.0	10.0	1.9	F
GROUP 2	G2F1	F	9	4	1.0	8.1	3.8	M
	G2F2	F	17	11	0.9	8.5	3.2	F
	G2F3	F	10	5	0.8	9.6	2.4	M
	G2M1	M	7	2	1.0	7.7	3.9	M
GROUP 3	G3M1	M	23	11	1.0	9.3	4.3	F
	G3M2	M	10	2	0.8	9.8	2.7	F
	G3M3	M	11	3	1.0	9.7	2.8	M
	G3M4	M	9	3	1.0	8.7	3.6	F
	G3M5	M	11	3	1.0	8.7	3.1	F
	G3F1	F	13	6	0.8	9.2	2.2	F
	G3F2	F	19	10	1.0	7.8	4.6	F
ALL	Mean ± ES		13.3 ± 1.3	5.5 ± 1.0	0.9 ± 0.1	9.0 ± 0.2	3.2 ± 0.2	
	CV (%)		40.0%	69.6%	14.4%	7.7%	22.9%	

^1^ conception rate = Mean number of foals produced by the mare per year.

**Table 3:** Foal weaning and inter-group variations: age of foals and duration of the dry period.

Variables	Study Group 1	Study Group 2	Study Group 3
Mean age at weaning (in months)	9.3 ± 0.2	8.5 ± 0.4	9.0 ± 0.3
[Min-Max] (in months)	[8.8–10.0]	[7.7–9.6]	[7.8–9.8]
CV (%)	4.8%	9.7%	7.7%
Duration of the dry period (in months)	3.0 ± 0.3	3.3 ± 0.3	3.3 ± 0.3
[Min-Max] (in months)	[1.9–3.7]	[2.4–3.9]	[2.2–4.6]
CV (%)	23.3%	20.2%	26.1%
